# GLYNAC AND THE AGING MOUSE HEART: SEX-RELATED DIFFERENCES AND OUTCOMES

**DOI:** 10.1093/geroni/igae098.0414

**Published:** 2024-12-31

**Authors:** Katarzyna Cieslik, Aude Angellini, Grecia Garcia Marquez, Marta Fiorotto, George Taffet

**Affiliations:** Houston Methodist Hospital, Houston, Texas, United States; BCM, Houston, Texas, United States; BCM, Houston, Texas, United States; BCM, Houston, Texas, United States; Houston Methodist Hospital, Houston, Texas, United States

## Abstract

Aging is associated with increased reactive oxygen species (ROS) resulting from an imbalance in antioxidant defenses and a rise in proinflammatory milieu. Glutathione (GSH) is a naturally occurring antioxidant which concentration is reduced in aging. Studying hearts of 21 month old male and female mice, we found that the enzymes of the GSH pathway synthesis were not downregulated however, supplementing a diet with GSH precursors (termed GlyNAC) for 3 months, improved cardiac diastolic function (measured longitudinally) and exercise performance in males. A much smaller GlyNAC effect on cardiac function and exercise was seen in females. Unbiased cardiac proteome mass spectrometry (MS) analysis validated by protein expression and functional assays demonstrated that a GlyNAC-supplemented diet led to an increase of Ndufb8 expression (a subunit of the mitochondrial respiratory chain complex), and higher enzymatic activities for CPT1b and CrAT, two carnitine acyltransferases, critical to cardiomyocyte fatty acid metabolism. Crosslinking and expression of fibrillar collagens is directly linked with extracellular matrix (ECM) stiffness, and although diastolic function was improved with GlyNAC, no significant change in fibrillar collagen was detected in the hearts of GlyNAC-fed old males. MS analysis determined that GlyNAC increased fibromodulin (Fmod) expression, which can inhibit collagen fibril formation. In summary, these data suggest that there are phenotypic sex-dimorphism differences in old mice and that these differences may affect the response to pharmacological and diet interventions, including GlyNAC. Furthermore, a simple diet intervention has been proven to ameliorate age-related cardiac diastolic dysfunction in old males.

